# Supplementary material for: The causal relationship between the human gut microbiota and pyogenic arthritis: a Mendelian randomization study
Source: Front Cell Infect Microbiol. 2024 Nov 26;14:1452480. doi: 10.3389/fcimb.2024.1452480 (PMC11629706; doi:10.3389/fcimb.2024.1452480)
Supplement: Supplementary file 1 [file DataSheet1.zip › mendelian test/ebi-a-GCST90027838.csv_leave_one_out.pdf]

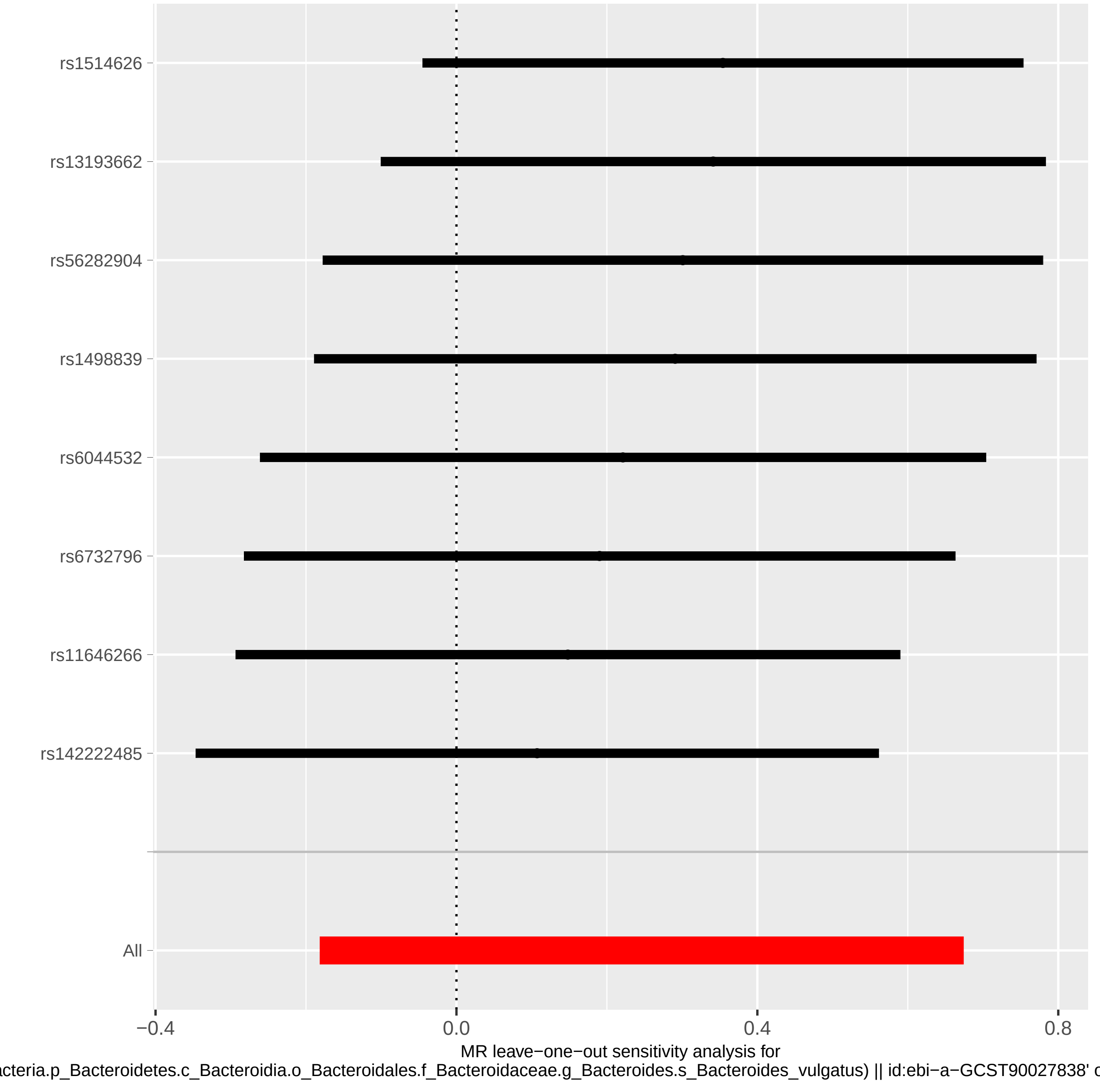

rs1514626
rs13193662
rs56282904
rs1498839
rs6044532
rs6732796
rs11646266
rs142222485
All
−0.4
0.0
0.4
0.8
MR leave−one−out sensitivity analysis for
acteria.p_Bacteroidetes.c_Bacteroidia.o_Bacteroidales.f_Bacteroidaceae.g_Bacteroides.s_Bacteroides_vulgatus) || id:ebi−a−GCST90027838' o
